# Red-shift of spectral sensitivity due to screening pigment migration in the eyes of a moth, *Adoxophyes orana*

**DOI:** 10.1186/s40851-017-0075-6

**Published:** 2017-08-30

**Authors:** Aya Satoh, Finlay J. Stewart, Hisaharu Koshitaka, Hiroshi D. Akashi, Primož Pirih, Yasushi Sato, Kentaro Arikawa

**Affiliations:** 1 Laboratory of Neuroethology, SOKENDAI (The Graduate University for Advanced Studies), Shonan Village, Hayama, 240-0193 Japan; 2 Division of Tea Research, Institute of Fruit Tree and Tea Science, National Agriculture and Food Research Organization (NARO), Kanaya, 428-0039 Japan

**Keywords:** Compound eye, Screening pigment migration, Spectral sensitivity, Phototaxis, Action spectrum

## Abstract

**Background:**

We have found that the spectral sensitivity of the compound eye in the summer fruit tortrix moth (*Adoxophyes orana*) differs in laboratory strains originating from different regions of Japan. We have investigated the mechanisms underlying this anomalous spectral sensitivity.

**Methods:**

We applied electrophysiology, light and electron microscopy, opsin gene cloning, mathematical modeling, and behavioral analysis.

**Results:**

The ERG-determined spectral sensitivity of dark-adapted individuals of all strains peaks around 520 nm. When light-adapted, the spectral sensitivity of the Nagano strain narrows and its peak shifts to 580 nm, while that in other strains remains unchanged. All tested strains appear to be identical in terms of the basic structure of the eye, the pigment migration in response to light- and dark-adaptation, and the molecular structure of long-wavelength absorbing visual pigments. However, the color of the perirhabdomal pigment clearly differs; it is orange in the Nagano strain and purple in the others. The action spectrum of phototaxis appears to be shifted towards longer wavelengths in the Nagano individuals.

**Conclusions:**

The spectral sensitivities of light-adapted eyes can be modeled under the assumption that this screening pigment plays a crucial role in determining the spectral sensitivity. The action spectrum of phototaxis indicates that the change in the eye spectral sensitivity is behaviorally relevant.

## Background

The spectral sensitivity of photoreceptors is determined by several factors [1]. The primary factor is the absorption spectrum of the cell’s visual pigment, which absorbs light energy and activates the phototransduction cascade. Second, various screening pigments in the eye tissue modify the spectral profile of the incoming light before it is absorbed by the visual pigments. These filtering effects of screening pigments can significantly affect the photoreceptor spectral sensitivity [2–4]. Third, the optical structure of the eye, including that of the photoreceptive organelles, often acts as a spectral filter that affects the spectral sensitivity of photoreceptors [5].

Similar to the human retina, which separately expresses three color opsins in three types of cone photoreceptor cells, insect compound eyes are typically furnished with three classes of spectral photoreceptors, each expressing the opsin of a short- (S or UV), middle- (M or blue) or long-wavelength (L or green) absorbing visual pigment. These spectral receptors provide the physiological basis of insect trichromacy [6–8]. However, the number of visual pigment opsins and, consequently, the number of photoreceptor types varies considerably among species. Multiplication of opsins is rather common [9–11], and is most likely associated with the species’ color vision ability. For example, a *Papilio* butterfly that expresses three L opsins (L1–3) in addition to one S and one M opsin has tetrachromatic color vision based on the UV, blue, green and red sensitive photoreceptors; the green and red receptors express L2 and L3, respectively [12, 13].

Color vision is widespread in diurnal insects such as bees and butterflies, but also plays a crucial role in some nocturnal species [14, 15]. In the course of studying the mechanism and evolution of color vision at low light levels, we encountered an unexpected phenomenon in the summer fruit tortrix moth, *Adoxophyes orana*. *A. orana* is a widespread pest species that feeds on a variety of plants, with a particular preference for apples. Management of this species without using chemical insecticides would be beneficial for protecting apple crops worldwide. We have therefore initiated a study on their vision to explore possible means of reducing crop damage using light. Here we report that spectral sensitivity in this species varies between strains originating from different regions of Japan. We further detail the spectral sensitivity variation and elucidate the underlying mechanisms by applying a number of techniques including electrophysiology, anatomy, molecular biology, model simulation and behavioral analysis. We conclude that the *A. orana* eye is a peculiar variant of compound eye [16], where pigment migration upon light adaptation modifies not only the eye’s absolute sensitivity, but also its spectral characteristics.

## Methods

### Animals

We used the summer fruit tortrix moth, *Adoxophyes orana*, from four laboratory strains derived from about 100 wild-caught individuals (larvae, pupae) from Nagano, Niigata, Akita, and Aomori prefectures in eastern Japan. These strains have been kept at Kanaya Division of Tea Research, NARO Japan for more than 170 generations (~20 years). Ten to 20 newly emerged females were mated with the males from the original population, and were allowed to lay eggs. Hatched larvae were reared on an artificial diet of Insecta LFS (NOSAN Corp., Japan) at 21 °C under a light regime of 14 h light:10 h dark (14L10D). Adults of subsequent generations were outcrossed to keep the genetic variability of the strain. For the present experiments, newly emerged virgin adults were kept in plastic containers with water-soaked filter paper in groups of ~10 individuals under a 12L12D light regime and never fed.

### Histology

We processed the eyes of male adults of Niigata and Nagano strains for light and electron microscopic histology. The light-adapted eyes were fixed under regular room light. The dark-adapted eyes were dissected under dim red light (white LED covered with a red polycarbonate filter, Supergel #25, Rosco), and immersed in the fixative in the dark for at least 30 min, and then further processed under regular light as follows.

For electron microscopy (EM), isolated eyes were prefixed in 1% paraformaldehyde and 4% glutaraldehyde in 0.1 M sodium cacodylate buffer (CB, pH 7.4) at 4 °C overnight. The samples were then washed in CB, postfixed in 2% OsO_4_ in CB for 2 h at room temperature, followed by *en-bloc* staining in 2% UrAc in 50% EtOH for 2 h at room temperature. The postfixed eyes were dehydrated in a graded series of acetone, infiltrated with propylene oxide, and embedded in Quetol 812. Ultrathin sections, cut with a diamond knife, were stained with uranyl acetate and lead citrate, and observed in a transmission electron microscope (H7650, Hitachi, Tokyo, Japan). For light microscopy (LM), isolated eyes were fixed in 2% paraformaldehyde and 2.5% glutaraldehyde in CB for 30 min on ice. After washing with CB, the samples were dehydrated and embedded in Quetol 812. The tissues were cut into 10 μm sections and observed with a light microscope (BX51, Olympus, Tokyo, Japan).

### Molecular cloning of visual pigment opsin

The heads were isolated from nine male individuals of three strains (1 Aomori, 4 Niigata and 4 Nagano individuals), and total RNA was separately extracted from each head using TRIzol reagents (Invitrogen) and purified using RNeasy mini kit (Qiagen). Complementary DNA (cDNA) was synthesized using a high-capacity reverse transcription kit (Applied Biosystems). We designed degenerate primers to amplify partial L opsin sequences by PCR. The forward and reverse primer sequences were as follows: forward (5′- ttgaagcttcarttyccnccnatgaaycc-3′) and reverse (5′- cgaattcggrttrtanacigcrttngcytt −3′). The PCR products, which contain all seven trans-membrane regions, were then cloned in T-Vector pMD20 (TaKaRa), and 3–5 clones from each individual were analysed to determine the nucleotide sequences of *A. orana* L opsin genes.

We compared the sequences of *A. orana* opsins among the different strains. To predict the sites of amino acid substitutions and their contributions to possible change in the absorption spectra, we aligned these sequences to the amino acid sequences of the squid visual pigment [17].

### Electrophysiology

The spectral sensitivity of the compound eye was determined by recording electroretinograms (ERG). A moth was fixed with beeswax onto a plastic stage. Chlorided silver wire inserted in the abdomen served as the reference electrode. The tip of a glass micropipette filled with physiological saline was inserted into the compound eye. After leaving the mounted sample for several minutes in the dark Faraday cage, the eye was stimulated with a series of monochromatic flashes of 100 ms duration separated by 10 s intervals. The light was provided by a 500 W xenon arc lamp through a series of narrow-band interference filters ranging from 300 to 700 nm (half band-width = 10–14 nm, Asahi Spectra, Japan). We swept through the wavelengths from short to long and then repeated the sequence in reverse. Such pairs of bidirectional recordings were made several times with different light intensities. We then measured the response-stimulus intensity (V-log I) function over a 4 log unit intensity range at the wavelength that gave the maximum response, and converted the spectral responses into spectral sensitivities using the V-log I function. The ERG responses were recorded through a MEZ-7200 preamplifier (Nihon Kohden, Tokyo, Japan) connected to a computer via a MP-150 AD converter (BIOPAC, USA).

To directly compare spectral sensitivities in dark- and light-adapted states, we took moths during the dark period (3 h into the scotophase), inserted the electrode under dim red light, and kept the animal in the dark Faraday cage for 1 h. We then recorded ERGs using monochromatic lights at 15 s intervals. After completing our recordings in the dark, we applied strong white illumination for 5 min to light-adapt the eyes, and then took further measurements from that individual.

### Imaging microspectrophotometry (IMSP)

Imaging spectrophotometry was performed similarly to that described previously [18]. Briefly, we used unstained 10 μm-thick sections of eyes prepared for LM histology. Light from a xenon lamp source was filtered through interference filters (360 to 700 nm, 20 nm intervals) and fed through a quartz light guide (1 mm, NA 0.50, Thorlabs). The light was focused on the sample by a custom condenser with a LUCPlanFl 20 × air objective lens (NA 0.45, Olympus, Japan). The light transmitted was collected with a LUCPlanFl 60× air objective lens (NA 0.85, Olympus) and imaged on a monochromatic camera (FL3-U3-13S2M-CS, FLIR, Canada). We took 16-bit image stacks of the tissue, imported them to Fiji/ImageJ and converted into floating point precision transmittance stacks *T*(*x,y,λ*). An empty part of the microscope slide was used to obtain the bright reference values. The stacks were aligned using the StackReg plugin. In each sample, we selected three regions in the image where pigments of the secondary pigment cells were densely packed (distal), and other three regions in the middle part of the photoreceptor layer where pigments in the photoreceptor cells were present but pigments of secondary pigment cells were scarce (proximal). The absorbance of each region, *A*(*λ*), was calculated from the average pixels transmittance value as *A*(*λ*) = −log_10_ {*T*(*λ*)}_*x,y*_. For exemplary regions of the distal and proximal pigments, see Fig. 2d.

### Pigment adaptation model

The simplest case of ERG-determined spectral sensitivity with three visual pigments (S, M and L) can be modeled as a weighted sum of template absorption spectra:

1 $$ {S}_0\left(\lambda \right)={\sum}_{n=S,M,L}{f}_n{R}_n\left(\lambda \right) $$

where *R*_n_(*λ*) are the templates defined by their peak parameters *λ*_max,n_ [19] and *f*_n_ are their relative contributions. This model assumes a single intensity-response function, ignoring metarhodopsin absorption and waveguide effects [20]. Screening pigments function as simple optical filters absorbing some light before it reaches the visual pigment, especially in the light-adapted state. We estimated the transmittance of the pigments *T*_i_ as:

2 $$ {T}_i\left(\lambda \right)=\mathit{\exp}\left(-{k}_i{A}_i\left(\lambda \right)\right) $$

where *k*_i_ and *A*_i_(*λ*) are respectively the effective density and the pigment absorbance spectrum. Then the light-adapted spectral sensitivity *S*(*λ*) is:

3 $$ S\left(\lambda \right)=c{S}_0\left(\lambda \right){T}_D\left(\lambda \right){T}_P\left(\lambda \right) $$

where *c* accounts for general attenuation, and *T*_D_ and *T*_P_ are the transmittances of the distal and proximal pigments. Combining eqs. (2) and (3) then yields

4 $$ S\left(\lambda \right)=c{S}_0\left(\lambda \right)\mathit{\exp}\left(-{k}_D{A}_D\left(\lambda \right)\right)\mathit{\exp}\left(-{k}_P{A}_P\left(\lambda \right)\right) $$

The pigment absorption spectra, *A*_i_(*λ*), were heuristically modeled by fitting a sum of two Gaussian functions to the normalized absorbance spectra:

5 $$ {A}_i={p}_1\mathit{\exp}\left\{-{\left(x-{\mu}_1\right)}^2/2{\sigma}_1^2\right\}+{p}_2\mathit{\exp}\left\{-{\left(x-{\mu}_2\right)}^2/2{\sigma}_2^2\right\} $$

For parameters, see Table 1. This and all subsequent model fitting were performed using the non-linear least-squares algorithm *nlsLM* from the “minpack.lm” package for R.

**Table 1:** Parameters for screening pigments

Strain	Pigment	*A*_1_	*μ*_1_ (nm)	*σ*_1_ (nm)	*A*_2_	*μ*_2_ (nm)	*σ*_2_ (nm)
Niigata	distal	0.59	156.3	422.9	0.35	525.0	61.5
	proximal	0.51	403.9	108.7	0.40	629.4	92.5
Nagano	distal	0.59	483.0	113.6	−0.19	604.0	66.4
	proximal	0.39	445.1	132.7	0.10	514.9	46.7

Parameters: $$ {A}_i={p}_1\mathit{\exp}\left\{-{\left(x-{\mu}_1\right)}^2/2{\sigma}_1^2\right\}+{p}_2\mathit{\exp}\left\{-{\left(x-{\mu}_2\right)}^2/2{\sigma}_2^2\right\} $$

### Behavioral experiments

We compared the action spectra of phototaxis among different strains, to determine whether the unusual eye sensitivity pattern of the light-adapted Nagano strain (see Results) affects its behavior. We used partially light-adapted moths of the Nagano and Niigata strains, because fully light-adapted moths were not active at all in our experimental setup. We kept the moths on a 14L10D light regime with ca. 1 lx illumination throughout the scotophase. All moths were tested during the last 2 h of the scotophase, because male *A. orana* are thought to be most active around this time for mating [21]. The state of the eyes of the partially light-adapted moths was confirmed by LM histology.

The experimental arena (W x H x D = 23 x 16 x 16 cm) was made from black cardboard with a window measuring 2 × 2 cm on one wall of the cage. The window was covered with a sheet of tracing paper acting as a light diffuser. Inside the cage, we made a 1.5 cm wide border around the window with sticky flypaper tape (Lifelex, Japan). Monochromatic stimuli were provided by a 100 W xenon arc lamp through a series of narrow-band interference filters, with peak transmittance at 540, 560, 600, and 680 nm (Asahi Spectra). The light intensity was attenuated with ND filters. The photon flux of each monochromatic light was measured at the window’s inner surface using a radiometer (Model 470D, Sanso, Japan). Temperature and humidity were kept at approximately 22 °C and > 50%, respectively. We released 8–14 males in the arena and initially kept the arena dark for 1 min. Then the window was illuminated by a monochromatic light from outside for 20 min, after which we counted the number of individuals trapped on the sticky area around the window. Between one and six trials were performed for each lighting condition.

The response-stimulus intensity data were fitted to a sigmoidal function, *R*/*R*_*max*_ = *I* ^*n*^/(*I* ^*n*^ + *K* ^*n*^), where *I* is the stimulus intensity, *R* is the proportion of males captured on the trap, *K* is the stimulus intensity required for 50% capture and *n* is the slope of the sigmoid. We plotted the reciprocal of *K* versus wavelength to obtain the action spectrum of phototactic behavior.

## Results

### Anatomy

**Fig. 1:**
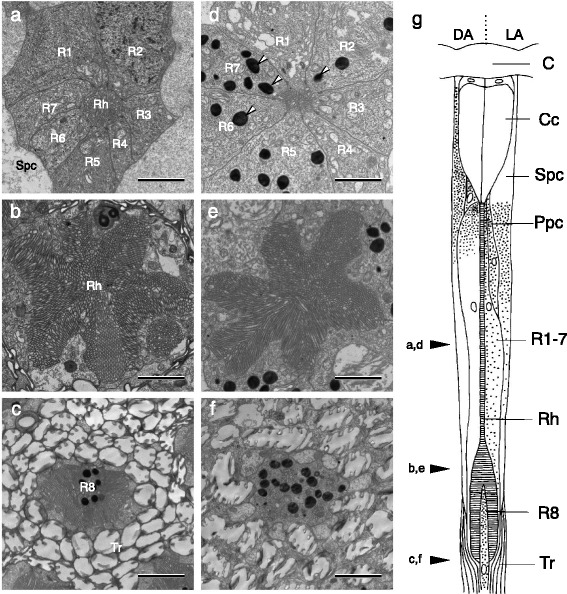
Ommatidial structure of male *Adoxophyes orana*. Transverse sections of the rhabdom (Rh) region in dark-adapted (**a**–**c**) and light-adapted (**d**–**f**) eyes. The depths at which these sections were cut are indicated in the diagram (**g**) by three black arrowheads on the left. **a** Distal rhabdom (Rh) of a dark-adapted ommatidium. Seven photoreceptors (R1–7) are evident. Cell bodies of photoreceptors and secondary pigment cells (Spc) are free of pigment granules. **b** Proximal rhabdom. **c** Basal rhabdom. Pigmented cell is the eighth and basal photoreceptor, R8. The ommatidia are surrounded by numerous tracheoles (Tr). **d** Distal rhabdom of a light-adapted ommatidium. Pigment granules (white arrowheads) exist in the region close to the rhabdom. **e** Proximal rhabdom. **f** Basal rhabdom. **g** Diagram of an ommatidium in dark- (left) and light- (right) adapted states. C, cornea; Cc, crystalline cone; Ppc, primary pigment cell. Scale bars = 2 μm

Electron microscopy of the photoreceptor layer indicated that a single ommatidium of *A. orana* bears eight photoreceptor cells, R1–8. Under the cornea and crystalline cone, photoreceptors R1–7 form a single rhabdom whose diameter is about 1 μm (Fig. 1a, d). The rhabdom broadens and becomes star-shaped as it proceeds proximally (Fig. 1b, e). At the bottom of the rhabdom, the heavily pigmented R8 photoreceptor appears in the center of the ommatidium, which is surrounded by numerous tracheoles (Fig. 1c, f).

The structure of ommatidia changes due to dark- and light-adaptation, with prominent movement of screening pigment granules. Figure 1g shows a diagram of one ommatidium, the left half being dark-adapted and right half light-adapted. When dark-adapted, the pigment granules in the secondary pigment cells migrate distally, surrounding the crystalline cone. The pigment granules in the primary pigment cells as well as the photoreceptor cells also move distally, making the middle of the photoreceptor layer almost transparent. The pigment granules move proximally upon light adaptation. The crystalline cone region becomes free of pigment, while the photoreceptor layer becomes pigmented. The pigment granules of the photoreceptors also migrate radially towards the rhabdom, resting within 1 μm of the rhabdom border (Fig. 1d, e). The pigment in this region would absorb the boundary wave of light propagating along the rhabdom. Probably because of the extensive pigment migration, the sizes of the photoreceptor cell bodies and primary pigment cells change in the distal portion of the retina (Fig. 1a–g).

**Fig. 2:**
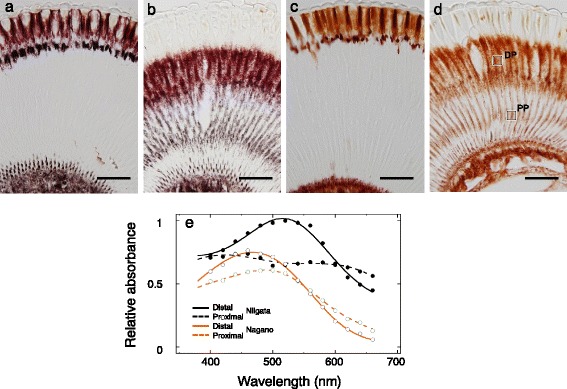
Light micrographs of longitudinal sections of the eye. **a** Dark-adapted male from the Niigata strain. **b** Light-adapted, Niigata. **c** Dark-adapted, Nagano. **d** Light-adapted, Nagano. **e** Absorption spectra of distal pigmented (DP) and proximal pigmented (PP) regions, which are exemplarily indicated by squares in **d**. Circles indicate IMSP data and the lines indicate heuristically modeled fits (Table 1). Average of the measurements from 3 points in each strain. Scale bars = 50 μm

The pigment migration is even more evident using light microscopy (Fig. 2a-d). The photoreceptor layer is transparent in dark-adapted eyes (Fig. 2a, c), while it is pigmented in light adapted eyes (Fig. 2b, d). Another striking feature is the color appearance of the pigments. The pigment is either dark purple (distal pigment) or dark brown (proximal pigment) in the Niigata strain (Fig. 2a, b), but orange in the Nagano strain (Fig. 2c, d). The pigment color of the Akita and Aomori individuals appears similar to that of the Niigata individuals (data not shown). We measured the absorbance spectra of the pigments by IMSP. Distal pigments correspond mainly to the pigment granules in the secondary pigment cells, and proximal pigments are those in the photoreceptor cells (Fig. 2e). The orange pigment of the Nagano strain mostly absorbs wavelengths below 600 nm, while the dark brown pigment in photoreceptor cells of Niigata strain absorbs light more evenly throughout the UV-visible spectrum.

### Visual pigments of *A. orana*

**Fig. 3:**
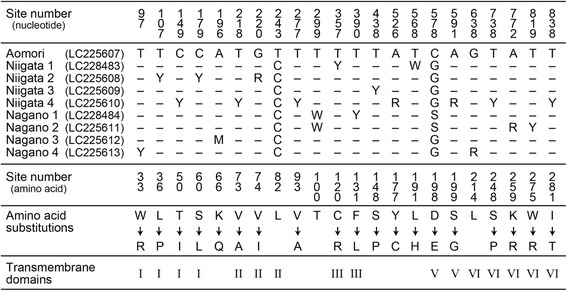
Variations of nucleotide sequences detected in *A. orana* L opsins from nine individuals of three populations with data accession numbers. Partial L opsin nucleotide sequences of one Aomori, four Niigata, and four Nagano individuals were determined. Nucleotide variations and the resulting amino acid substitutions were shown with the site numbers. Dashes (−) indicate the nucleotides identical to the Aomori individual. Site numbers and trans-membrane domains were deduced based on alignment to the squid visual pigment. One-letter abbreviations for the nucleotides were following: Y for C and T, M for A and C, W for A and T, R for A and G, S for C and G

We determined partial nucleotide sequences (816 bp) of a long wavelength-type visual pigment opsin (L opsin), covering all seven transmembrane regions. Figure 3 shows the sequence data from nine individuals of *A. orana*. These individuals were taken from the Aomori (*n* = 1), Niigata (4) and Nagano strains (4). Figure 3 shows 22 sites of nucleotide variations that we found among the individuals. Twenty one of them were heterozygous, and 19 correspond to non-synonymous amino acid substitutions. Although more than half of these variations are located in the transmembrane domains, there was no non-synonymous mutation that differed systematically between the Niigata and Nagano strains. It is thus likely that the absorption spectra of different strains’ L opsin are identical. We chose to analyze the L opsin as it plays the dominant role in determining the spectral sensitivity to wavelengths above 500 nm; the structure of the blue and UV opsins remains to be studied.

### Spectral sensitivity of the compound eye of *A. orana*

**Fig. 4:**
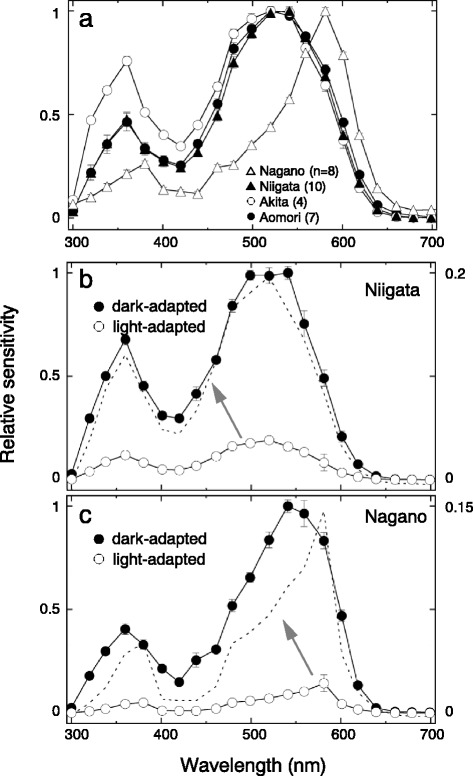
ERG-determined compound eye spectral sensitivities of male *Adoxophyes orana*. **a** Averaged light-adapted spectral sensitivities of individuals from Nagano (*n* = 8), Niigata (10), Akita (4) and Aomori (7) strains. **b** Dark- and light-adapted spectra recorded from the same Niigata individuals (*n* = 2), to measure the change in absolute sensitivity in the light-adapted state. The dotted spectrum shows the light-adapted spectrum expanded vertically (scale on the right). **c** Same as **b**, for the Nagano strain (*n* = 1)

An electroretinogram (ERG) represents a collection of photoreceptor responses from a large region of the eye around the inserted electrode. Figure 4a shows ERG-determined spectral sensitivities of individuals from four strains originating from Nagano, Niigata, Akita and Aomori prefectures. We measured the sensitivities of individuals during their photophase when the eyes were light-adapted (Fig. 2b, d). The spectral sensitivity of the Nagano individuals is narrow and peaks at 580 nm, while other strains’ sensitivities are broader, with their peaks at around 520 nm.

The eye sensitivity changes upon dark- and light-adaptations. Figure 4b and c each shows a set of spectra based on the spectral sweeps before (supposedly dark-adapted) and after (supposedly light-adapted) intense white illumination lasting 5 min. In both Niigata (Fig. 4b) and Nagano (Fig. 4c) strains, light-adaptation lowers the overall sensitivity about one log unit. (Dotted lines are replots of the light-adapted spectra on the expanded axis on the right). Light-adaptation narrows the spectral sensitivity and shifts its peak in the Nagano individual (Fig. 4c).

### Contribution of screening pigments

**Fig. 5:**
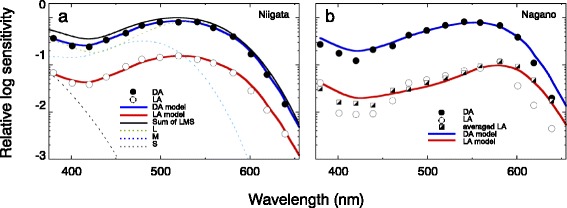
Modelled spectra of dark- (blue line) and light- (red line) adapted eyes with the recorded spectral sensitivities shown in Fig. 4 **b** and **c** (circles). Model fits were performed over the wavelength range for which pigment spectra were available. For coefficients, see Table 1. **a** Niigata strain. Dotted spectra show visual pigment templates (grey, S; blue, M; green, L) with their relative peak values according to *f*_i_. The solid black line denotes the sum of these three spectra, i.e. the sensitivity in the absence of screening pigment. **b** Nagano strain. For illustration (because the light-adapted spectrum is based on a single individual and may therefore be noisy), half-filled squares show the averaged spectral sensitivity shown in Fig. 4 **a** with open triangles, scaled to the appropriate amplitude

To investigate the mechanism underlying the red-shifted spectral sensitivity in the light-adapted Nagano individuals, we applied our pigment adaptation model (see Methods). We used the within-individual dark- and light-adapted spectral sensitivity data shown in Fig. 4b and c for the model fitting. We first fitted the dark-adapted *S*(*λ*) for the Niigata individuals (Fig. 4b, filled circles) with *λ*_max,n_, *f*_n_ and *k*_i_ as free parameters (Table 2). The resultant *λ*_max_ for S, M and L visual pigments (and their weightings *f*_n_) were 344 nm (0.16), 481 nm (0.48) and 533 nm (1.25), respectively, which are shown individually and summed in Fig. 5a. The difference between the visual pigment summation (black line) and the complete model (blue line) corresponds to the pigment contribution, which appears to be minor: the contributions of the distal and proximal pigments, *k*_D_ and *k*_P_, were 0 and 1.2, respectively. Because the *λ*_max_ values of the three visual pigments appear biologically plausible [22], we used these *λ*_max_ and *f*_n_ values in subsequent calculations, based on the assumption that the visual pigment molecules (Fig. 3) and their expression pattern are identical between the Niigata and Nagano strains.

**Table 2:** Calculated normalization and pigment contribution factors

Strain	State	*c*	*k*_D_	*k*_P_
Niigata	DA	1.0	0.0	1.2
	LA	1.0	0.3	4.2
Nagano	DA	2.0	3.1	0.0
	LA	2.0	0.0	9.8

*Abbreviations*: *c* general attenuation factor, *k*_D_ effective density of distal pigment, *k*_P_ effective density of proximal pigment

We then estimated *k*_D_ and *k*_P_ as free parameters for the other conditions. The normalization factor *c* was included to account for the separate normalization of the two strains, but was kept constant across the two adaptation states of each strain. The best-fit curves are shown in Fig. 5. The model fitting well explained both the light-adapted Niigata spectrum (Fig. 5a, red) and the dark-adapted Nagano spectrum (Fig. 5b, blue). The model deviates somewhat from the light-adapted Nagano spectrum (Fig. 5b, open circles), but the fit appears more reasonable when compared with the averaged spectral sensitivity (half-filled squares, same spectrum shown in Fig. 4a), suggesting that the poor fit is (at least partially) due to noisy data from a single individual. It is clear that the contribution of the proximal screening pigment is much stronger in the light-adapted states, while that of the distal screening pigment is minor (Table 2).

The fitting results indicate that the spectral sensitivity shift in light-adapted Nagano individuals is mainly due to the screening effect of the photoreceptor pigment that migrates towards the rhabdom upon light adaptation.

### Behavioral experiments

**Fig. 6:**
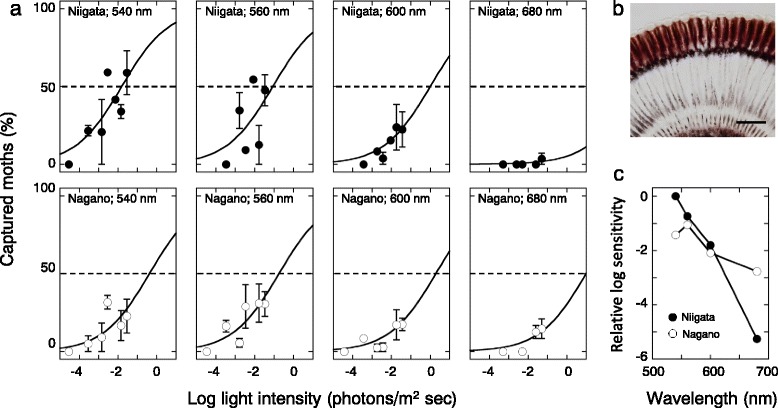
**a** Phototactic response to four monochromatic lights for Niigata and Nagano strains. Percentage of captured individuals is plotted against the light intensity. Eight to 14 individuals were used in each trial. Mean ± standard error. **b** A longitudinal section of a partially light-adapted eye of a Niigata individual. **c** Action spectra of the Niigata (filled circles) and Nagano (open circles) individuals

Figure 6a shows the results of the phototaxis experiments. We tested partially light-adapted (see Methods) individuals from the Niigata and Nagano strains at four wavelengths of light, varying intensity over 3 log units. Vertical axes represent the percentage of moths captured on the sticky surface around the illuminated window, which we use as a measure of attraction efficacy. Eye sections of the partially light-adapted individuals (Fig. 6b) show that the photoreceptor pigment has indeed migrated towards the rhabdom. We calculated the sensitivity to each stimulus (based on the light intensity required to elicit a 50% response), and Fig. 6c shows the resulting action spectra. Moths of the Niigata strain exhibit highest sensitivity at 540 nm among the wavelengths we tested, whereas those of Nagano strain are maximally sensitive at 560 nm.

## Discussion

### Perirhabdomal pigment as a dynamic spectral filter

*Adoxophyes orana* appears at first glance to have a typical superposition-type eye with a large unpigmented zone and pronounced pigment migration upon dark and light adaptation (Fig. 1). The unpigmented region of superposition eyes is called a clear zone, and has no photoreceptive function in itself. However, the EM histology of the unpigmented zone of *A. orana* reveals that there each ommatidium has an elongated thin rhabdom whose diameter is about 1 μm. The distal tip of the thin rhabdom abuts directly onto the proximal end of the crystalline cone, which is a characteristic feature of apposition-type compound eyes. Such an optical arrangement seems fairly common among moths [23–27], and may be advantageous for “an animal that must cope with the change in light intensity that occurs during the twilight period, as *Adoxophyes* does” [25].

Incident light excites the ommatidial photoreceptors by activating visual pigments in the rhabdom, which acts as an optical waveguide. A fraction of light propagating along the optical waveguide actually travels outside of it as the boundary wave. The waveguide optical model predicts that the pigment surrounding a thin rhabdom functions as an optical filter by absorbing the boundary wave [20]. The present modeling supports the notion that the photoreceptor screening pigment indeed attenuates the eyes’ sensitivity, although we have not attempted to model the details of the waveguide effect. In the Niigata strain individuals, the overall eye sensitivity drops about one log unit when light-adapted, while keeping the spectral sensitivity unchanged. In the Nagano strain, on the other hand, light adaptation shifts the spectral sensitivity towards longer wavelengths in addition to decreasing overall sensitivity (Fig. 4). Because our molecular biology study has suggested that the absorption spectra of visual pigments are identical among strains (Fig. 3), the difference in adaptation effects may be attributable to differences in the absorption spectra of photoreceptor screening pigments (Fig. 2).

Perirhabdomal pigments have been shown to play a lateral filtering role [5] in some animals, including mantis shrimps [2] and butterflies [28]. In these diurnal species however, the pigments do not migrate much upon light and dark-adaptation, so the pigment effect is rather constant. The pigment migration observed here (Fig. 2) usually takes place between day and night under the control of biological clocks [29, 30]. The Nagano individuals therefore experience daily changes in eye spectral sensitivity, which may affect their visual behavior especially around dusk. Circadian changes in the eyes’ spectral sensitivity have also been reported in a few twilight-active fireflies [31] and flies [32], which presumably experience large changes in light intensity while they are active around dusk.

### Phototactic response

The action spectra of the phototactic response roughly mirror the eye’s spectral sensitivity (Fig. 6). In the Niigata strain, sensitivity is highest at 540 nm, and decreases as the wavelength becomes longer. We could not determine the peak of the spectrum in the present study, but it most likely exists somewhere around 520 nm [33]. The action spectrum of the Nagano strain peaks at 560 nm. This probably corresponds to the long wavelength shift of the eye spectral sensitivity in this strain. However, the action spectrum is less shifted than one might expect based on the eye sensitivity results (c.f. Figs. 4b and 6c), likely because the individuals we used were not fully light-adapted. Partial light adaptation means that fewer pigment granules migrate to where they can efficiently absorb the boundary wave, thus reducing the degree of spectral shift. Because *A. orana* is most likely a crepuscular species, partial light adaptation is probably a rather common situation in the wild.

### Evolution of the orange pigment

Photostable screening pigments in the eye fundamentally function as pupils, which attenuate light influx upon light adaptation [34, 35]. In some cases, colorful perirhabdomal pigments should be regarded as serving a modified function: creating a greater variety of spectrally dissimilar photoreceptors in order to improve color discrimination ability [2, 36]. The migrating pigment in *A. orana* clearly functions as a pupil, as seen in the Niigata strain, without changing spectral sensitivity (Fig. 4b). In the Nagano strain however, we see a combined effect of pupil and spectral filter (Fig. 4c), whose biological significance is not yet known. We at least know that the Nagano case is rather exceptional. It could be that pigments are evolutionarily labile and therefore important for environmental adaptation.

The Nagano strain we analyzed here originates from eggs laid by 10–20 females that were captured as larvae in the field in July 1998. Unfortunately, there are no reliable records on the eye pigment and spectral sensitivity of the first generation individuals. We also observed another established laboratory strain from Nagano prefecture, but from a different region. We found that their eye pigments and spectral sensitivities were all similar to those of the Niigata individuals (data not shown). Therefore, it is possible that the observed orange pigment is due to the so-called founder effect, where the variation in pigment colors existed in a minority of individuals in the original wild population. The orange genotype may somehow have been dominant early in the strain lineage and thus become fixed. Alternatively the variation may have been absent from the original population and arisen once the strain was in captivity. Comprehensive analysis of genomic DNA or eye mRNA could provide some clues to identify the mutation(s) responsible for the pigment color change. A more extensive survey of field-captured individuals would provide information about the possible biological function of the orange pigment, the visual ecology of *A. orana*, and potentially also about effective techniques for region-specific integrative pest management using light [37].

## Conclusions

The screening pigment in the compound eyes of Nagano strain individuals of *A. orana* is orange while that of other strains is purple. We conclude that the anomalous eye spectral sensitivity of the light-adapted Nagano individuals is due to the migration of the orange screening pigment. Although the evolutionary background of the orange pigment in the Nagano strain still remains unclear, the reported phenomenon implicates the necessity of region-specific strategies of integrative pest management.
